# Long-read sequencing reveals genomic landscape of structural variants in a major disease vector

**DOI:** 10.64898/2026.10.05.756236

**Published:** 2026-10-07

**Authors:** Remi N Ketchum, Anna W Trotter, Rafael J Vivero, Madelyn A Pivovarnik, Daniel R Schrider, Daniel R Matute

## Abstract

The southern house mosquito Culex quinquefasciatus is a major disease vector for multiple arboviruses, including West Nile virus and St. Louis encephalitis virus. Insecticides are widely used to control Cx. quinquefasciatus populations, but resistant mosquitoes have emerged in numerous locations across the globe, including the United States. Much progress has been made in recent years in identifying insecticide resistance (IR) mutations in various pest species. In most species, these studies have focused primarily on SNPs as they are readily detectable. However, several genomic structural variants (SVs) have been experimentally shown to impact IR in Drosophila melanogaster, and structural variation has been observed in natural populations of vector species at a few genes that may be relevant to IR (e.g., cytochrome P450s). Because most population genomic data are derived from short sequencing reads, which fail to detect a large fraction of SVs, the contribution of structural variants to the evolution of IR and other adaptations in vector species is likely to be underestimated in the absence of long-read sequencing data. Here, we present the first genome-wide assessment of structural variants in Cx. quinquefasciatus from wild populations in Puerto Rico and Zambia using PacBio long-read sequencing. We constructed high-quality genome assemblies for each and found a total of 259,046 SVs which consisted predominantly of insertions and deletions. We found that 154,071 of these variants contained or overlapped genes. Of these SVs, 5,225 intersected IR genes and 56 of these were predicted to result in changes to downstream protein function. Several SVs predicted to severely impact protein-coding genes also coincided with signatures of selective sweeps, highlighting their possible role as targets of selection. Many of these variants would have gone undetected using short-read approaches, underscoring the importance of long-read sequencing for comprehensive population genetic analyses of adaptation to pesticides and other strong selective pressures, not only in insects but across the tree of life.

## Full Text Availability

The license terms selected by the author(s) for this preprint version do not permit archiving in PMC. The full text is available from the preprint server.

